# The impact of a 12-week tele-exercise program on cognitive function and cerebral oxygenation in patients with OSA: randomized controlled trial—protocol study

**DOI:** 10.3389/fspor.2024.1418439

**Published:** 2024-09-06

**Authors:** Vasileios T. Stavrou, Konstantinos Pitris, Fofi Constantinidou, Tonia Adamide, Frangiskos Frangopoulos, Panagiotis Bargiotas

**Affiliations:** ^1^ Laboratory of Cardio-Pulmonary Testing and Pulmonary Rehabilitation, Respiratory Medicine Department, Faculty of Medicine, University of Thessaly, Larissa, Greece; ^2^ RespiHub, ONISILOS MSCA COFUND, University of Cyprus, Nicosia, Cyprus; ^3^ Department of Neurology, Medical School, University of Cyprus, Nicosia, Cyprus; ^4^ Department of Electrical and Computer Engineering, KIOS Research and Innovation Center of Excellence, University of Cyprus, Nicosia, Cyprus; ^5^ Department of Psychology, University of Cyprus, Nicosia, Cyprus; ^6^ Respiratory Clinic, General Hospital of Nicosia, Nicosia, Cyprus

**Keywords:** sleep apnea, brain health, tele-exercise, tele-rehabilitation, cognitive impairment

## Abstract

Obstructive sleep apnea (OSA) is associated with a number of adverse health effects, particularly on brain health. Chronic sleep disruption caused by OSA can adversely affect cognitive health. Exercise is recommended as a non-pharmacological intervention for patients who are intolerant to continuous positive airway pressure (CPAP) and has been shown to have beneficial effects on brain health and cognitive function. This protocol aims to investigate the effects of a 12-week tele-exercise program on cognitive function and specific parameters of brain activity, including brain metabolism and oxygenation, in patients with OSA. The project aims to demonstrate the multidimensional relationship between exercise, cognition, and brain oxygenation/metabolism. Our local ethics committee has approved the study. Our population sample (Group A, OSA with cognitive impairment (CI) and tele-exercise; Group B, OSA with CI and no tele-exercise; Group C, OSA without CI and no tele-exercise) will undergo assessment both before and after a 12-week tele-exercise intervention program. This assessment will include a comprehensive battery of subjective and objective assessment tests. Data will be analyzed according to group stratification. We hypothesize a beneficial effect of tele-exercise on sleep and cognitive parameters, and we are confident that this study will raise awareness among healthcare professionals of the brain health benefits of exercise in patients with low compliance to CPAP treatment. The protocol of our manuscript entitled "The impact of a 12-week tele-exercise program on cognitive function and cerebral oxygenation in patients with OSA: Randomized Controlled Trial -Protocol Study" has been registered on ClinicalTrials.gov with ID NCT06467682.

## Graphical Abstract

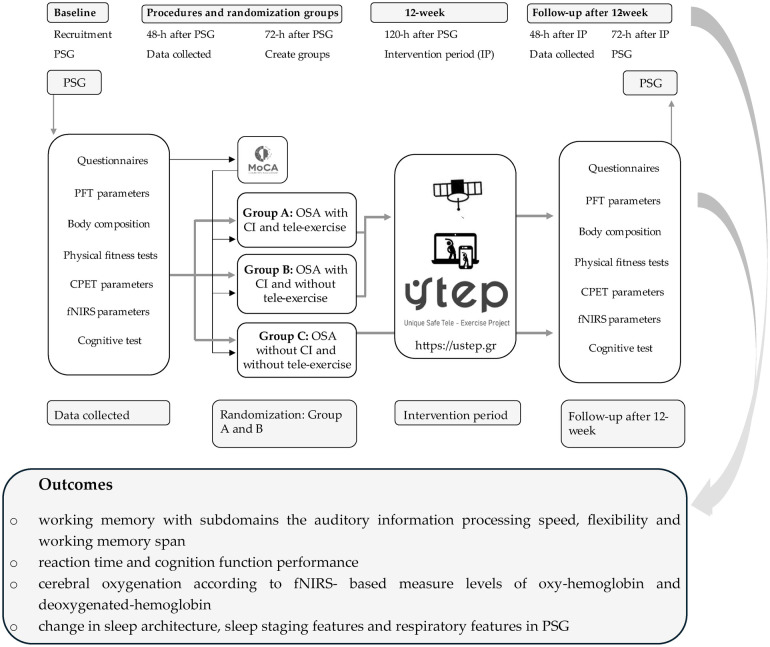

CI, cognitive impairment; CPET, cardiopulmonary exercise testing; fNIRS, functional near-infrared spectroscopy; h, hour; IP, intervention period; MoCA, Montreal cognitive assessment; OSA, obstructive sleep apnea; PFT, pulmonary function test; PSG, polysomnography study; USTEP, unique safe tele-exercise project.

## Background

Obstructive sleep apnea (OSA) is characterized by repeated episodes of obstruction of the upper airway during sleep causing intermittent hypoxia, sleep fragmentation, hypercapnia, and sympathetic hyperactivity (1). These factors contribute to various adverse health effects, notably impacting brain health. Hyperarousability in OSA can negatively impact sleep in the macro- and micro-architecture and sleep continuity, both of which are important players in neurogenesis, brain plasticity, alertness, and the processes of memory formation and consolidation (2). Thus, chronic sleep alterations caused by OSA can negatively impact cognitive health (2). Both sleep fragmentation and intermittent hypoxia affect brain structure and function, increasing susceptibility to neurodegenerative diseases (3). The prevalence of OSA is increasing as it is conjoined with obesity and tends to elude clinicians’ attention, with definitive diagnosis achieved in only 10% of the population. Approximately 80% of OSA patients report reduced performance at work (4), while 40% of the risk of dementia can be attributed to modifiable risk factors such as physical inactivity (5).

Exercise provides a wide range of benefits to the general population by improving cardiorespiratory and metabolic profiles (6). Exercise is recommended as a low-cost, easy-to-administer, non-pharmacological intervention with beneficial effects on brain health and cognitive function, mainly by improving sleep architecture, enhancing the neurovascular oxygenation process and cerebral oxygenation, reducing sympathetic overactivity, and improving vascular function at rest and during exercise or mental stress (6, 7). In addition, studies have shown that exercise can mitigate various aspects of brain deterioration, such as reduced blood flow and lack of important factors [e.g., brain-derived neurotrophic factor (BDNF)]. These factors play a key role in nourishing the brain's neurons and promoting the growth and development of new neurons and synapses. Based on classical theories (8), physical exercise can be considered an enhancing environmental factor that promotes neuroplasticity (increasing blood and oxygen flow to the brain, stimulating neuron growth, and increasing nerve conduction). In addition, exercise has beneficial biological and psychological effects on the brain, cognitive function (9), and sleep (10) and triggers potent neuroplastic phenomena, partly mediated by epigenetic mechanisms (9). Therefore, long-term exercise produces significant and lasting benefits such as improved cerebral neurovascular dynamics, cerebrovascular function, cognition, and neuroplasticity in various brain regions, parameters related to cerebral oxygenation and metabolism, which can be assessed by functional near-infrared spectroscopy (fNIRS) (11, 12). In addition, exercise intensity correlates with cognitive functions such as memory improvement after 12-week programs of both aerobic and resistance exercise (13, 14). The effectiveness of different exercise programs has not been well established, and some studies suggest that supervised vs. self-selected programs may have similar outcomes (15, 16). In addition, the types of exercise programs in patients with OSA have been addressed in the literature (6). However, there is a significant dropout rate from face-to-face exercise programs, especially if they last several weeks, due to high cost, difficulty, and/or lack of access to rehabilitation centers (6). Tele-exercising could be a key to overcoming this important limitation. However, data on tele-exercise is scarce.

This protocol aims to investigate the effects of a 12-week tele-exercise program on cognitive function and specific parameters of brain activity, including brain metabolism and oxygenation, in patients with OSA. The project aims to highlight the multidimensional relationship between physical exercise, cognition, and brain oxygenation/metabolism.

## Methods

This study is a randomized controlled trial and has been approved by the National Bioethics Committee of Cyprus (EEBK/EP 2023/60), the State Health Services Organization of Cyprus (05.34.001.002/44/23), and ClinicalTrials.gov (NCT06467682). Written informed consent will be obtained from patients in accordance with the tenets of the Declaration of Helsinki (17) and the regulations of the European Parliament and Council of the European Union (18).

### Study population

This study will include newly diagnosed patients with OSA, and participants will be recruited from the Sleep Apnea Outpatient Clinics and Sleep–Wake Outpatient Clinics of the Nicosia General Hospital. We will perform a screening cognitive test via the Montreal cognitive assessment (MoCA) questionnaire to identify individuals with cognitive impairment (CI). Patients with OSA and CI will be randomized into two groups: The OSA group with CI who will undergo the tele-exercise program (TE) (Group A) and the OSA group with CI who will not undergo the TE (Group B). A third matched group (Group C) of OSA patients without cognitive impairment will act as a second control group. Group A will perform a tele-exercise program on the Unique Safe Tele-Exercise Project platform (https://ustep.gr), for 12 weeks. All patients who will be enrolled in our study will not receive continuous positive airway pressure (non-CPAP) treatment (delayed therapy) (Graphical Abstract).

The inclusion criteria for this study will be the following: age ≥18 to ≤70 years, apnea–hypopnea index (AHI) ≥15 events/h, no contraindications (e.g., unstable angina during the previous month, myocardial infarction in the previous month, resting heart rate >120 bpm, systolic blood pressure >180 mmHg, and diastolic blood pressure >100 mmHg) for the 6 min walk test (6MWT) (19, 20), body mass index (BMI) <40 kg/m^2^, no daily physical workload, no active self-reported symptoms (fatigue and dyspnea) (21, 22, 23), and pregnancy. Patients with pre-existing musculoskeletal disability were excluded because their condition may affect maximal exercise capacity (24). All participants should be able to read and understand Greek.

### Assessment

All participants will be asked to sign a consent form prior to any study-related assessment. The baseline assessment will be conducted before the randomization of participants into groups, and the same assessment protocol will be used immediately after the 12-week intervention period (IP).

The study-related assessments include the following. (a) *Polysomnography study* (PSG): all patients will undergo a home-based sleep study using a portable PSG device (SOMNOtouch RESP, SOMNOmedics, Germany) for the investigation of sleep quality and the classification of sleep apnea severity (25). (b) *Questionnaires*: (i) Montreal cognitive assessment (MoCA) to investigate the cognitive status and classify participants into groups (26), (ii) two-part trail making test (TMT A and *Β*) to assess shifting attention and selective attention (27), (iii) Pittsburgh sleep quality index (PSQI) to assess the quality and patterns of sleep (28), (iv) Epworth sleepiness scale (ESS) to assess daily sleepiness levels (29), (v) Karolinska sleepiness scale (KSS) to assess the situational sleepiness (30), and (vi) work ability index (WAI) to investigate the ability to return to work without restrictions (31). (c) *Pulmonary function test*: standard spirometry testing (32) followed by the diffusing capacity of the lungs for carbon monoxide (MasterScreen-CPX, VIASYS Healthcare, Germany) according to ATS/ERS guidelines (33) and respiratory muscle strength (Airofit PRO, Copenhagen, Denmark) (34). (d) (i) *Anthropometric characteristics*: anthropometric characteristics such as body height, chest circumference in maximal inhalation and exhalation, neck circumference, and waist–hip ratio will be recorded (Seca 700 and 201, Hamburg, Germany) (35); (ii) *body composition*: body mass, muscle mass, percentage of body fat, visceral fat score, total body water, etc. will be recorded via bioelectrical impedance analysis (Tanita MC-980, Arlington Heights, IL, USA) (35). (e) *Physical fitness test*: we will use physical fitness tests to assess exercise capacity via 6MWT (19) and 30 s sit-to-stand test (36). Before, at the end of tests, and at the first minute of recovery, blood pressure, arterial O_2_ saturation, and heart rate will be recorded, and patients will self-assess for dyspnea and lower limb fatigue via CR-10 Borg scale (37). The isometric power will be recorded via a handgrip strength test (KFORCE, Kinvent Estonia) (23). (f) *Cardiopulmonary exercise testing (CPET) parameters*: we will record the cardiopulmonary (i.e., oxygen consumption, carbon dioxide production, ventilation, tidal volume, and breath frequency end-tidal partial pressure of carbon dioxide and oxygen) and metabolic parameters, via portable device COSMED K5 (38) before, during, and at the first minute of recovery of each physical fitness test (6MWT and 30 s sit-to-stand test). (g) *Cognitive test*: we will use the FitLight Trainer (Sports Corp., Ontario, Canada) integrated into wireless unit touch and motion sensors. All participants will perform tests of a pre-planned motion structure to assess perceptual skills, executive functions, and brain sensory functions such as reactivity, movement accuracy, and visuospatial capacities (39, 40). (h) *Functional near-infrared spectroscopy (fNIRS) parameters*: we will use the fNIRS device (BIOPAC Systems, Inc. Goleta, CA, USA) to assess and record cerebral oxygenation-oxygenated and deoxygenated hemoglobin (fNIRS) before, during, and after physical fitness and cognitive tests (41, 42).

Study-related outcomes include the following. (a) *PSG study outcome*: for this aim, we will compare several sleep/wake questionnaires, and various polysomnographic quantitative and qualitative parameters at baseline and 12-week follow-up will be assessed, e.g., change in sleep architecture, sleep staging features, and respiratory features in PSG between baseline and 12-week follow-up. (b) *Questionnaire*s *on the cognitive outcome*: (i) verbal episodic memory based on subdomains of the encoding process and immediate recall, free and cued recall, and recognition and delayed recall; (ii) score in a serial verbal learning task, with control of encoding and recall, according to the modified procedure of Grober and Buschke (43); and (iii) working memory with subdomains the auditory information processing speed, flexibility, and working memory span. (c) *CPET parameters outcome*: to investigate the extent to which tele-exercise improves cardiopulmonary metabolic parameters in patients with OSA at baseline and 12-week follow-up in participants with non-CPAP treatment. (d) *fNIRS outcomes*: cerebral oxygenation according to fNIRS-based measure levels of oxyhemoglobin (HbO) and deoxygenated hemoglobin (deoxy-Hb). For this aim, we will compare cerebral oxygenation values fNIRS at baseline and following a 12-week follow-up in the abovementioned three groups of patients.

All the above assessments will be performed at baseline and 12-week follow-up.

### Intervention protocol

The 12-week TE program will be performed and supported by the USTEP platform, with each patient taking part in three training sessions per week with a 60 min duration period per training session. There will be personalized training sessions: (i) warm-up and warm-down (5 min each) with mobility and proprioception exercises and respiratory exercises, (ii) aerobic exercise (30 min) with continuous outdoor walking and/or home-based with intensity on 90%–120% of HR_peak_ calculation according to HR_peak_ during 6MWT and self-reported feeling of dyspnea and leg fatigue (all participants of the tele-exercise program will use wearable-based tracking to record the covered distance and assess the cardio-oxygenation), and (iii) multijoint strength exercises (20 min) to improve the strength of upper and lower limbs on 70%–80% intensity calculation according to kilograms and repetitions (combined of HR_peak_) (this will include handgrip strength test and 30 s sit-to-stand). All tele-exercise program participants will use wearable-based tracking to assess the cardio-oxygenation. Adherence to the tele-exercise program will be recorded through wearable-based tracking, and the dedication rate will be recorded through Google Analytics, which is linked to the USTEP platform.

### Statistical analysis

For the sample size calculation of this study, a power of 85% and a confidence interval of 95% were adopted (G*Power software 3.1), with an estimated type 1 error value of 5%, given the lack of previous studies investigating the effects of a tele-exercise program in patients with OSA. We determined a sample size of 20 patients for each group. The Kolmogorov–Smirnov test will be used to assess data normality for continuous variables. ANOVA analysis of variance will be applied to compare the groups before and after the intervention period. Relationships between continuous variables will be assessed via Spearman's rho and Pearson's R correlation coefficients for non-parametric and parametric variables, respectively. Differences between cutoff strata will be assessed via the independent samples *t*-test or Mann–Whitney *U*-test where appropriate. Analysis of fNIRS data will be based on a boxcar general linear model and a custom MATLAB (MathWorks, Natick, MA, USA) script (44). A *p*-value of <0.05 will be considered statistically significant for all tests. The IBM SPSS 21 statistical package (SPSS Inc., Chicago, IL, USA) will be used for all statistical analyses.

## Discussion

This study is designed to explore the impact of a 12-week tele-exercising program on the cognitive profile as well as on specific parameters of brain function and sleep architecture in OSA patients without CPAP treatment. In addition, this study aims to highlight the multidimensional relationship between physical exercise, cognition, brain oxygenation/metabolism, and sleep quality.

### Expected research results

In the context of our protocol, we will record and evaluate the effects of tele-exercising as a complementary treatment for OSA. Using a standardized assessment process at the clinical level and through exercise capacity tests, we will draw conclusions that will correlate with possible changes in the profiles of patients with OSA and related health indicators. At the same time, tele-exercising will empower patients to experience the benefits of physical activity, particularly structured exercise, fostering a healthier lifestyle, and ameliorating comorbidities related to sleep disorders. OSA is known to be a disease that is affected, among other things, by increased body mass and low physical activity which, in combination with the pathophysiology of the disease, leads patients to comorbidities. Poor sleep quality, as recorded through the multirecord sleep study, is highly correlated with cognitive decline in patients with OSA. Tele-exercising has been positively associated with reducing patients’ anxiety and depression, improving cardiopulmonary function, and enhancing quality of life. In addition, tele-exercising creates and develops skills due to the lack of supervision resulting in participants exercising for longer periods of time without being detrained.

### Significance of the protocol

The originality of the present protocol lies in the fact that through the intervention of a period of tele-exercising and the measurement of control parameters, possible changes will be investigated both in the profile of patients with OSA and certain health indicators. The results of the present research protocol are estimated to contribute to a more complete understanding of the pathogenic mechanisms underlying the association between physical exercise, cognition, and brain oxygenation/metabolism. Finally, tele-exercising may contribute to the creation of intervention protocols in the pulmonary rehabilitation of patients with OSA who are unable to participate in organized pulmonary rehabilitation centers due to economic, social, and geographical difficulties. The successful implementation of the use of tele-exercising through the form of the platform (https://ustep.gr/) will be able to support even more patients with OSA to reduce their symptoms and improve their health.
